# Yeast communities of secondary peat swamp forests in Thailand and their antagonistic activities against fungal pathogens cause of plant and postharvest fruit diseases

**DOI:** 10.1371/journal.pone.0230269

**Published:** 2020-03-16

**Authors:** Petlada Satianpakiranakorn, Pannida Khunnamwong, Savitree Limtong

**Affiliations:** 1 Department of Microbiology, Faculty of Science, Kasetsart University, Bangkok, Thailand; 2 Academy of Science, The Royal Society of Thailand, Bangkok, Thailand; Universita degli Studi di Pisa, ITALY

## Abstract

Secondary peat swamp forest (PSF) arise by degradation of primary PSF as a result of fire and human activities. Yeasts diversity of Kuan Kreng (KK) and Rayong Botanical Garden (RBG) PSF, which are two secondary PSF in southern and in eastern Thailand, respectively, were investigated. Yeasts were isolated from soil and peat soil by the dilution plate and enrichment techniques. From six samples collected from KK PSF, 35 strains were obtained, and they were identified based on the sequence analysis of the D1/D2 region of the large subunit (LSU) rRNA gene 13 species in 12 genera, and one potential new species of the genus *Galactomyces* were detected. Thirty-two strains were obtained from six samples collected from RBG PSF and 26 strains were identified as 13 known yeast species in 11 genera, whereas six strains were found to represent two potential new species of the genera *Papiliotrema* and *Moesziomyces*. Among yeast strains isolated from KK PSF, the number of strains in the phylum Ascomycota and Basidiomycota were equal, whereas there were slightly fewer strains in Ascomycota than in Basidiomycota among the strains obtained from RBG PSF. The yeast strains were evaluated for their antagonistic activities against fungal pathogens which cause rice diseases (*Fusarium moniliforme*, *Helminthosporium oryzae*, *Rhizoctonia solani*, *Curvularia lunata* and *Pyricularia grisea*) and postharvest disease of fruits (*Phytophthora palmivora*, *Lasiodiplodia theobromae* and *Colletotrichum gloeosporioides*). Twelve strains of seven species were found to be antagonistic yeast strains. *Starmerella kuoi* DMKU-SPS13-6, *Hanseniaspora lindneri* DMKU ESS10-9 and *Piskurozyma taiwanensis* DMKU-SPS12-2 capable to inhibit *R*. *solani* by 70.1–76.2%, *Wickerhamomyces anomalus* DMKU SPS6-1 and three *Rhodotorula taiwanensis* strains (DMKU SPS8-1, DMKU ESS9-3, DMKU SPS9-2) inhibited *C*. *lunata* by 69.8–71.9%, *Hanseniaspora lindneri* DMKU ESS10-9 and *Scheffersomyces spartinae* DMKU SPS9-3 inhibited *P*. *grisea* by 81.9–84.4% and four *Papiliotrema laurentii* strains (DMKU-SPS15-1, DMKU-ESS11-2, DMKU-ESS8-2, DMKU-ESS6-4) inhibited *P*. *palmivora* by 53.2–59.5%.

## Introduction

Peatlands are areas with a unique ecosystem where a layer of peat has naturally accumulated at the surface and are an important store of the world’s carbon resources [1]. This ecosystem covers 3% (4 million km^2^) of the earth’s surface area in 180 countries [2]. Peatland is characterized by the accumulation of partially decayed organic matter under waterlogged conditions and by having rainfall as its sole source of water [3, 4]. Ions in mineral soil may be transported into the organic layer in peat and increasing the acidity of the organic matter [5]. These conditions decrease the growth of microorganisms and the activity of hydrolytic enzymes during the degradation of the organic matter coexists with the swamp [6, 7]. Peatlands can be found in the arctic, boreal, temperate and tropical zones [4].

In the tropical zone, the peatland coexist with swamp forest, called tropical peat swamp forests (PSF), which are a unique and endangered ecosystem [8]. More than 60% of the world’s PSFs are in South-East Asia in Indonesia, Malaysia, Vietnam, Thailand and the Philippines [8]. In Thailand, the tropical PSFs are mainly located in the southern region (63,982 ha) [9]. Peat in the tropical PSFs is formed from incomplete decomposition of woody plant debris due to the acid and waterlogged conditions [10] and differs from peat formation in temperate and boreal regions, where peat originates mainly from mosses and herbs [11]. PSFs are classified based on the differences of their plant communities into two types: the primary PSF and the secondary PSF. The primary PSF is an area with a great diversity of plant species. The secondary PSF is primary PSF that has been compromised by drought, wildfires and land conversion, resulting in a decrease in the richness of plant species [12]. Both Kuan Kreng (KK) and Rayong Botanical Garden (RBG) PSFs are secondary PSFs. In 2009, the Thailand Institute of Scientific and Technological Research reported that, the predominant plants in KK PSF are *Lepironia articulate*, *Typha angustifolia*, *Cyperus imbricatus* and *Melaleuca cajuputi*, while the vegetation in the RBG PSF is dominated by *Melaleuca quinquenervia*, *Garcinia cowa*, *Calamus deerratus* and *Shorea bracteolate* [13]. Little is known about microbial diversity in peatlands; however, the microbial diversity in boreal and temperate peatlands has received more attention than that in tropical PSFs although to date, not many articles have reported the microbial composition of temperate and boreal peatlands [11, 14, 15]. Among these, a few articles have reported on yeast communities of peatlands in Canada and Russia [16, 17, 18, 19].

Rice (*Oryza sativa*) is an important economic crop and is one of the main Thai exports. It is widely cultivated in all regions of Thailand and occupies about half of the country's cultivated area (9.6 million hectares) [20]. One of the major problems in the cultivation of this crop is diseases caused by fungal pathogens, which greatly reduce productivity and quality. The major diseases of rice are bakanae, brown spot, dirty panicle, rice blast and sheath blight. Bakanae disease of rice caused by *Fusarium moniliforme* [21] has high economic impact in rice production in Asian countries [22]. Rice yield loss caused by this disease can be as high as 40% [23]. Brown spot disease caused by *Helminthosporium oryzae* is widespread in all rice production areas of the world [24]. In South and Southeast Asia, this disease causes 5% yield loss, but severely infected rice field can create up to 45% yield loss [25]. Sheath blight disease caused by *Rhizoctonia solani* is the second most important rice disease in the world [26]. In Thailand, this disease can cause of 25–35% yield losses [27]. Dirty panicle disease caused by *Curvularia lunata* is one of the most serious rice disease and causes of high yield losses [28]. Rice blast disease caused by *Pyricularia grisea* is also one of the major rice diseases. In Southeast Asia and South America, this disease was reported to cause 30–50% yield losses [29]. Mango (*Mangifera indica*) and durian (*Durio zibethinus*) are the two major fruit exports of Thailand. In 2017, Thailand was the leading exporter of fresh durian (490,488 tons), valued at 722.8 billion dollars, whereas, the total amount of mangoes exported was approximately 35,800 tons, valued at 52.0 billion dollars [20]. During the storage and transportation periods, postharvest diseases caused by fungal pathogens occur and result in considerable economic loss. *Lasiodiplodia theobromae* and *Colletotrichum gloeosporioides* are the causal agents of rot and anthracnose diseases, respectively, which are the two major postharvest diseases of mango [30]. These fungal pathogens infect the fruit mainly during ripening [31]. Fruit rot of durian caused by *Phytophthora palmivora* and can cause production losses of 20–25% [32]. The common strategy for fungal disease control is using chemical fungicides. However, there are many problems associated with their use, such as risks to human health and the threat of, environmental pollution and of the development of pathogen resistance [33]. For these reasons, the replacement of chemical fungicides with biocontrol agents (BCAs) could be considered as an alternative fungal disease management strategy.

Many yeast species have been reported to be capable of controlling plant pathogenic fungi. For example, *Metschnikowia pulcherrima* and *Pichia guilliermondii* have potential for controlling of bakanae disease of rice caused by *Fusarium fujikuroi* [34]. *Torulaspora globose* and *Saccharomyces cerevisiae* inhibit *Colletotrichum sublineolum* [35]. *Saccharomyces cerevisiae*, *Wickerhamomyces anomalus* and *M*. *pulcherrima* suppress bunch rot disease on grapes caused by *Botrytis cinerea* [36]. Yeasts of the genera *Candida*, *Barnettozyma*, *Hanseniaspora*, *Kazachstania*, *Metschnikowia*, *Pichia* and *Pseudozyma* are able to suppress anthracnose disease caused by *C*. *gloeosporioides* [37]. Although several yeast species have been reported as biocontrol agents of some diseases caused by fungal pathogens, new antagonistic yeasts. Therefore, the objectives of this study were to study the yeast diversity of KK and RBG PSFs based on culture-dependent approaches, using the dilution plate and enrichment techniques for yeast isolation and molecular taxonomy and phylogenetic analysis for identification, and to evaluate the antagonistic activities of yeasts against fungal pathogens that cause rice and postharvest fruit diseases.

## Materials and methods

### Sampling sites and soil sample collection

**Fig 1:**
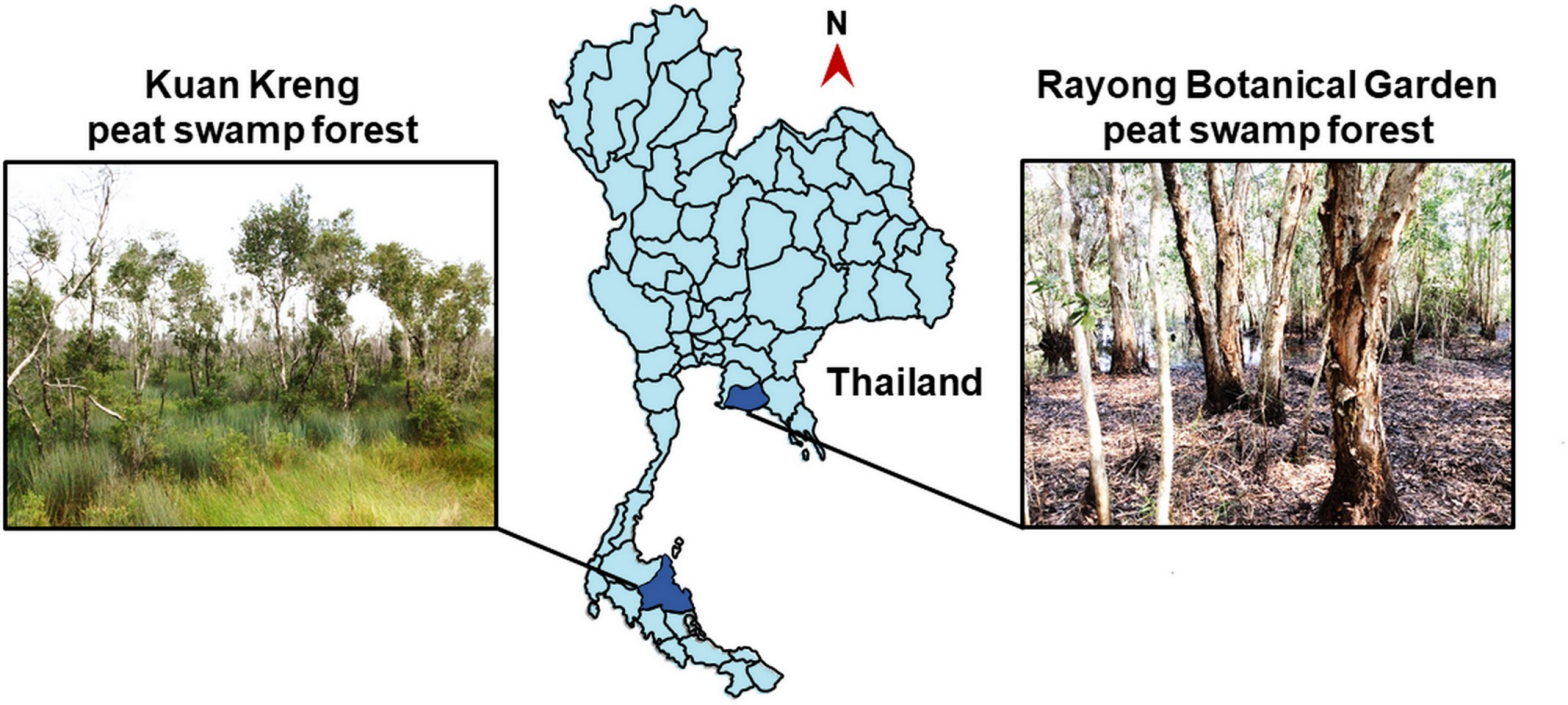
Maps of sampling sites. Sampling locations in Kuan Kreng and Rayong Botanical Garden peat swamp forests of Thailand.

Sample collection from KK and RBG PSFs (Fig 1), which were respectively permitted by Mr. Sawai Thongdam, chief executive, Subdistrict Administrative Organization Kreng, Cha-uat district, Nakhon Si Thammarat province, Thailand and Mr. Wachana Boonchai, head, Rayong Botanical Garden, Klaeng district, Rayong province, Thailand, were performed on 26 July 2017 and 29 January 2018, respectively. From each PSF, six soil samples were collected from six sampling sites. Soil sample was taken from 30–50 cm depth using an auger and put in a new plastic bag. The plastic bag was sealed and kept in an icebox during transfer to the laboratory. The samples were stored at 8°C until subjected to yeast isolation (no later than seven days after collection) and determination of peat characteristics.

### Physiochemical characteristic determination

Soil physicochemical characteristics were investigated by the method described by Boonmak et al. [38]. The pH of soil was measured from soil suspension (the soil:water ratio was 1:10) with a pH meter (EUTECH Instrument, CyberScan 1000, Singapore). Electrical conductivity (EC) of saturated soil extract was measured with an electrical conductivity meter (ATI Orion, model 162, Germany) [39]. One replicate was used for pH and EC determination. Organic matter was investigated by the titration method [40]. The N, P and K contents of soils were determined after wet digestion of soil sample by the Kjeldahl method [41] a spectrophotometer (Shimadzu, UV-1700. Japan), and an atomic emission spectrophotometer with a photomultiplier tube (PMT) detector at a burner high of 7 mm and air-acetylene was used at an acetylene flow rate of 2 L/min [42], respectively. Three replicates were performed for organic matter and N, P and K contents analyses.

### Yeasts isolation

Yeasts were isolated from each one of the 12 soil samples by the dilution plate and enrichment techniques as described by Boonmak et al. [38]. In brief, in the dilution plate technique, 11 g of soil sample was suspended in 99 ml of sterile 0.85% saline (NaCl) solution in a 250 ml Erlenmeyer flask and shaken on a rotary shaker at 150 rpm and room temperature (28±2°C) for 1 h. The soil suspension was three times serially ten-fold dilution diluted (1:10^2^ to 1:10^4^) with sterile 0.85% saline solution. Then 100 μl of each dilution (1:10 to 1:10^4^) were spread onto yeast extract-malt extract (YM) agar plates (0.3% yeast extract, 0.3% malt extract, 0.5% peptone, 1.0% dextrose and 2.0% agar) supplemented with 0.02% chloramphenicol and 0.025% sodium propionate. In the enrichment technique, YM broth supplemented with 0.02% chloramphenicol and 0.025% sodium propionate was used and incubation was carried out on a rotary shaker at 150 rpm and 25°C. After 2 days 100 μl of the culture broth was spread on a plate of YM agar supplemented with 0.02% chloramphenicol and 0.025% sodium propionate. After incubation at 25°C for 3–7 days, yeast colonies in each sample with different colony morphology were picked. Each colony was purified by cross streaking on an YM agar plate and repeated until pure culture was obtained. The purified yeast strains were suspended in YM broth supplemented with 10% (v/v) glycerol and maintained at -80°C.

### DNA extraction, amplification and sequencing

The genomic DNA extraction was performed as described by Limtong et al. [43]. The sequences of the gene encoding the D1/D2 region of the LSU rRNA gene and the internal transcribe spacer (ITS) region were amplified with the primers NL1 (5'-GCATATCAATAA GCGGGGAAAAG-3') and NL4 (5'-GGTCCGTGTTTCAAGACGG-3') [44] and the primers ITS1 (5'-TCCGTAGGTGAACCTGCGG-3') and ITS4 (5'-TCCTCCGCTTATTGA TATGC-3') [45], respectively. The PCR products were purified by using the TAINquick Midi Purification Kit (Tiangen Biotech, China) according to the manufacturer’s instructions. The purified products were sequenced by First Base Laboratories Sdn Bhd (Malaysia) using the PCR primers.

### Yeasts identification and phylogenetic analyses

The yeasts identification was based on molecular taxonomy and phylogenetic analysis of the D1/D2 region of the large subunit (LSU) rRNA gene sequence. The yeasts sequences were compared pairwise using BLASTn homology search [46]. For ascomycetous yeast identification, strains showing greater than 1% nucleotide substitutions in the D1/D2 region were considered to be different species and strains with 0–3 nucleotide differences were treated as conspecific species [44]. For basidiomycetous yeast, strains differing by two or more nucleotide substitutions were considered different species [47]. When necessary, the ITS sequences were also analyzed in order to assist the D1/D2-based identification. Phylogenetic analysis based on the sequences of the D1/D2 region of the LSU rRNA gene was used for confirming yeast identification by pairwise sequence similarity. The sequences of representative strains of individual species were aligned with type strains of their closest species using MUSCLE [48] provided within the MEGA version 7 software package. A phylogenetic tree was constructed from the evolutionary distance data using the general time reversible (GTR) model and the maximum-likelihood analyses performed with MEGA7 [49]. The confidences for the phylogenetic tree were estimated from bootstrap analysis (1000 replicates) [50]. The “potential new species” designation was used for the species that could not be identified by the above procedures.

### In vitro evaluation of yeasts antagonistic activity against fungal pathogens

Five fungal pathogens that cause rice diseases and three fungal pathogens that cause durian and mango fruit diseases after harvesting used in this study are shown in Table 1. Growth inhibition of a fungal pathogen by yeasts was evaluated by the dual cultivation technique as described by Rosa et al. [35] with slight modification. Potato dextrose agar (PDA) (Difco, USA) (20% potato infusion, 2.0% dextrose and 2.0% agar) in a Petri dish was streaked linearly with a loopful of active yeast cells (from a 2 day-old culture grown on YM agar at 25°C) 3 mm from one edge of the dish. After 2 days incubation at 25°C, a 5-mm-diameter mycelial plug of a 3 or 7-day-old culture of each fungal pathogen (grown on PDA agar at 25°C) was placed at the opposite edge of the dish. A PDA dish inoculated with only a mycelial plug of a fungal pathogen at one edge was used as a negative control. Three replicates were used for each combination yeast-pathogen and control. The inoculated dishes were incubated at 25°C for 3 days for *R*. *solani* and 7 days for other phytopathogenic fungi. Radial growth of fungal pathogen was measured and the inhibition (%) was calculated with the formula below [35].

$$\text{Inhibition of fungal growth}(\%)=\frac{\left(\text{radius of fungal colony cultured alone}-\text{radius of fungal colony cultured with yeast}\right)}{\text{radius of fungal colony cultured alone}}x100$$

**Table 1:** Fungal pathogens.

Fungal pathogen	Disease
Cause of rice diseases	
*Fusarium moniliforme* DOAC 1224	Bakanae
*Helminthosporium oryzae* DOAC 2293	Brown spot
*Rhizoctonia solani* DOAC 1406	Sheath blight
*Curvularia lunata* DOAC 2313	Dirty panicle
*Pyricularia grisea*	Blast
Cause of postharvest diseases	
*Phytophthora palmivora*	Rot in durian fruit
*Lasiodiplodia theobromae*	Rot in mango fruit
*Colletotrichum gloeosporioides*	Anthracnose in mango fruit

Fungal pathogens cause of plant and postharvest diseases used in this study.

### Statistical data analysis

Data of physiological characteristics and yeasts antagonistic activity against fungal pathogens were expressed as mean and standard deviation (SD). All statistical analyses were performed using the IBM SPSS statistics software version 22 for Windows. The significance difference at *P* < 0.05 of physiological characteristics data were analyzed by a one-way analysis of variance (ANOVA) according to Duncan’s multiple range test, whereas, of yeasts antagonistic activities data were analyzed by paired samples T test.

## Results

### Physiochemical characteristic of soils

The physiochemical characteristics of the 12 soil samples collected from the two secondary PSFs were analyzed and the results are shown in Table 2. The pH values of the soil samples collected from KK and RBG PSFs were acidic in the range of 3.1–3.7 and 4.7–5.7, respectively. The organic matter content of the soil samples collected from KK PSF varied considerably in the range of 6.45–93.64%. Therefore, based on the organic matter content as suggested by Huat et al. [51], only three samples: S03, S04 and S05, whose organic content was greater than 30%, were considered to be peat soils, whereas, the other samples were soils. The soil samples collected from RBG PSF appeared to contain low amounts of organic matter (2.14–10.51%), therefore, these samples were designated as soils.

**Table 2:** Sampling location and physicochemical characteristics.

Sample code	Location		pH	Electrical conductivity (dS/m)	Organic matter (%)^D^	Total NPK^D^			Available NPK			Type of sample
	Latitude	Longitude				N (%)	P (mg/kg)	K (mg/kg)	N (mg/kg)	P (mg/kg)	K (mg/kg)	
Kuan Kreng peat swamp forest, Cha-uat district, Nakhon Si Thammarat province, 27.2°C^A^^,^^B^, 111.2 mm^A^^,^^C^												
S01	7°55'21.3"N	100°06'59.3"E	3.56	0.66	11.95±0.06e	0.29±0.01b	226.90±18.91d	2,176.49±82.76de	26.05±4.10a	8.16±0.02a	29.79±1.02d	soil
S02	7°55'23.3"N	100°07'01.0"E	3.46	0.87	6.45±0.08bcd	0.12±0.00b	144.97±10.92c	2,124.43±64.26d	23.69±4.10a	17.90±0.07e	8.07±7.79a	soil
S03	7°55'21.9"N	100°07'03.4"E	3.16	1.45	46.34±1.32f	0.62±0.02c	252.12±21.84d	2,236.05±58.40e	52.11±4.10b	2.98±0.02b	21.45±0.58c	peat soil
S04	7°55'20.5"N	100°07'03.2"E	3.11	1.39	93.64±1.85h	0.59±0.01e	226.90±0.00d	1,233.11±29.44a	78.16±7.11c	1.15±0.00a	14.86±0.14b	peat soil
S05	7°55'19.7"N	100°07'01.6"E	3.50	0.68	72.72±2.56g	1.12±0.01d	560.96±21.84e	1,999.57±40.13c	71.05±0.00c	5.13±0.05c	55.76±0.36e	peat soil
S06	7°55'20.4"N	100°06'59.4"E	3.73	0.49	5.47±0.11bc	1.13±0.01b	220.60±71.59d	1,739.55±52.26b	28.42±0.00a	51.78±0.13f	10.40±0.24a	soil
**Rayong Botanical Garden peat swamp forest, Klaeng district, Rayong province, 27.7°C^A^^,^^B^, 230.0 mm^A^^,^^C^**												
SR01	12°39'05.4"N	101°32'43.0"E	5.73	0.02	2.14±0.04a	0.09±0.00a	27.11±0.00a	2,176.49±82.76de	26.05±4.10a	8.16±0.02d	29.79±1.02d	soil
SR02	12°38'41.5"N	101°33'40.4"E	4.75	0.05	8.13±0.02d	0.18±0.02a	60.41±5.77ab	2,124.43±64.26d	23.69±4.10a	17.90±0.07e	8.07±7.79a	soil
SR03	12°39'28.5"N	101°33'40.4"E	5.21	0.03	4.85±0.13b	0.15±0.02a	77.05±0.00b	2,236.05±58.40e	52.11±4.10b	2.98±0.02b	21.45±0.58c	soil
SR04	12°39'11.9"N	101°32'41.6"E	4.72	0.04	6.93±0.09cd	0.15±0.01a	53.75±5.77ab	1,233.11±29.44a	78.16±7.11c	1.15±0.00a	14.86±0.14b	soil
SR05	12°39'13.7"N	101°32'41.9"E	5.08	0.49	10.51±0.2e	0.20±0.00a	63.74±11.53ab	1,999.57±40.13c	71.05±0.00c	5.13±0.05c	55.76±0.36e	soil
SR06	12°40'03.6"N	101°33'23.8"E	5.15	0.03	6.96±0.11cd	0.17±0.01a	70.40±5.7ab	1,739.55±52.26b	28.42±0.00a	51.78±0.13f	10.40±0.24a	soil

Sampling location and physicochemical characteristics of soil samples in Kuan Kreng and Rayong Botanical Garden peat swamp forests.

Samples from Kuan Kreng peat swamp forest and Rayong Botanical Garden peat swamp forest were collected on 26 July 2017 and 29 January 2018, respectively.

^A^ Data from National Statistical Office, Ministry of Information and Communication Technology.

^B^ Average atmospheric temperature obtained in the particular month of the sampling area.

^C^ Average rainfall obtained in the particular month of the sampling area.

^D^ Each sample was determined in triplicate.

a, b, c, d, e, f, g and h indicated statistically significant (*P* < 0.05) intergroup difference

### Yeasts from Kuan Kreng peat swamp forest identified by molecular taxonomy and phylogenetic analyses

**Fig 2:**
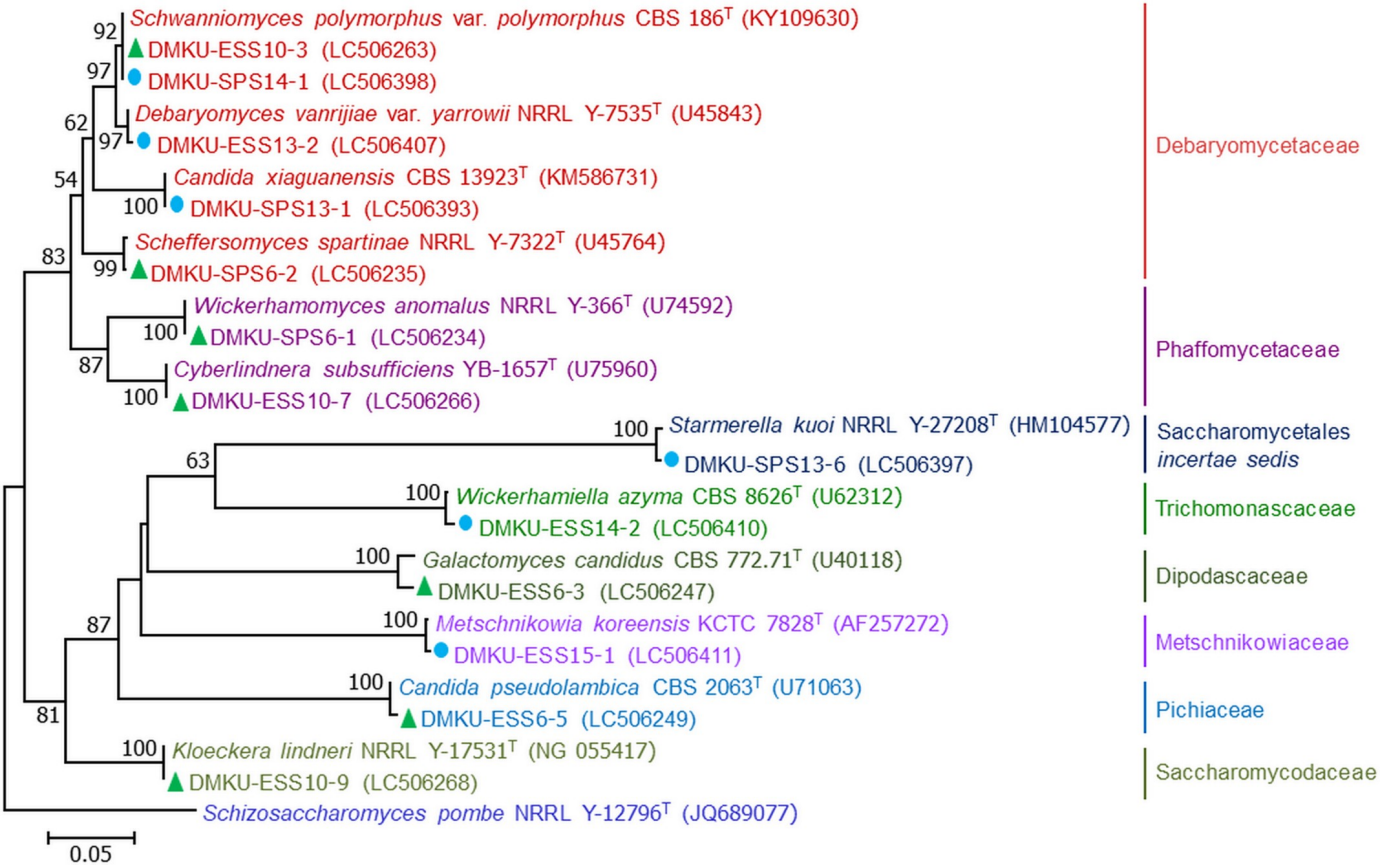
Phylogenetic placement of ascomycetous yeasts. Phylogenetic placement of known yeast species of Kuan Kreng (▲) and Rayong Botanical Garden (●) peat swamp forests belonging to the phylum Ascomycota and their closely related taxa based on the sequences analysis of the D1/D2 region of the LSU rRNA gene using maximum-likelihood method (GTR model). The names in bold type are representative strains from this study. Numbers on branches indicate percentages of bootstrap sampling (>50%), derived from 1000 samples. Bars indicate 0.05 substitutions per nucleotide position.

**Fig 3:**
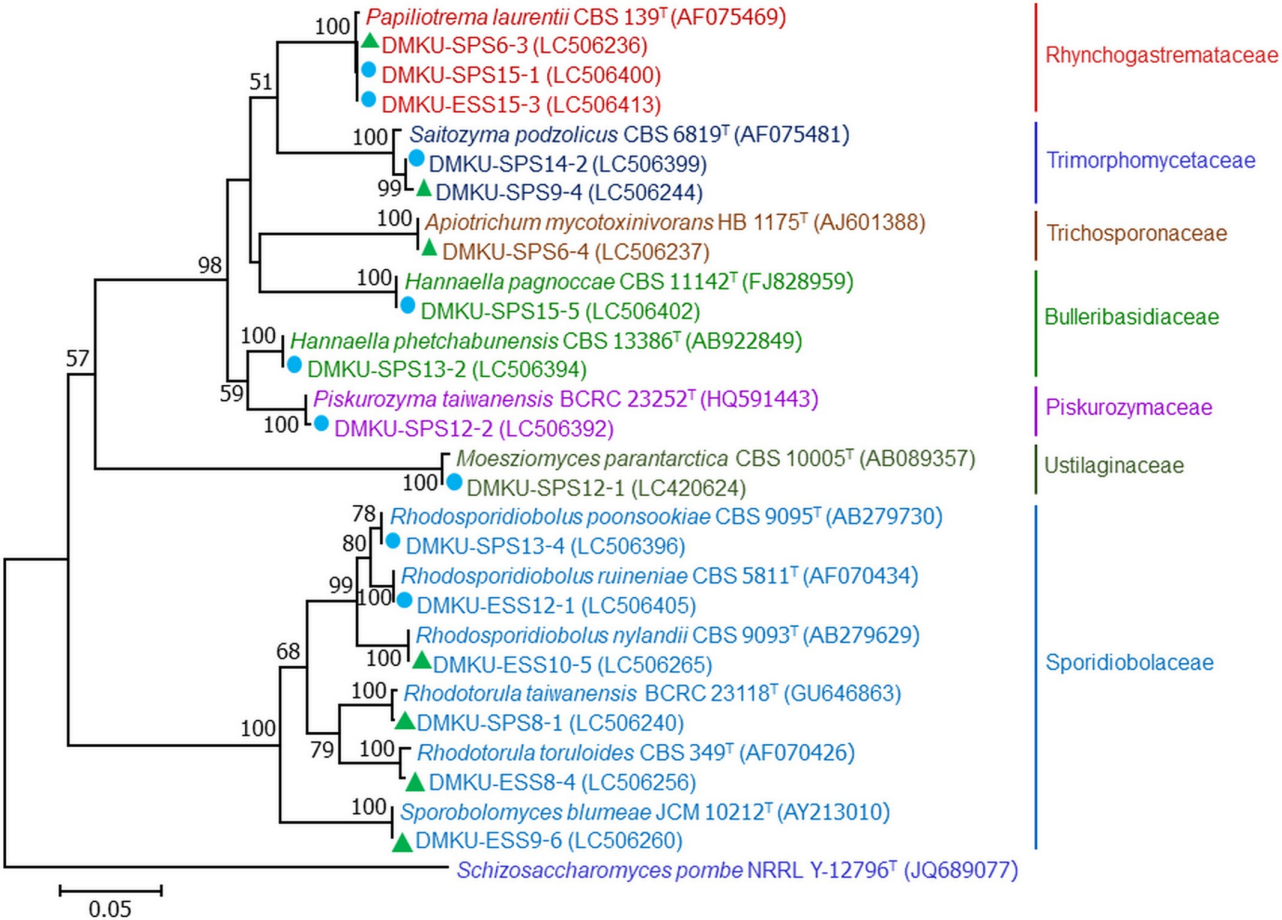
Phylogenetic placement of basidiomycetous yeasts. Phylogenetic placement of known yeast species of Kuan Kreng (▲) and Rayong Botanical Garden (●) peat swamp forests belonging to the phylum Basidiomycota and their closely related taxa based on the sequences analysis of the D1/D2 region of the LSU rRNA gene using the maximum-likelihood method (GTR model). The names in bold type are representative strains from this study. Numbers on branches indicate percentages of bootstrap sampling (>50%), derived from 1000 samples. Bars indicate 0.05 substitutions per nucleotide position.

**Fig 4:**
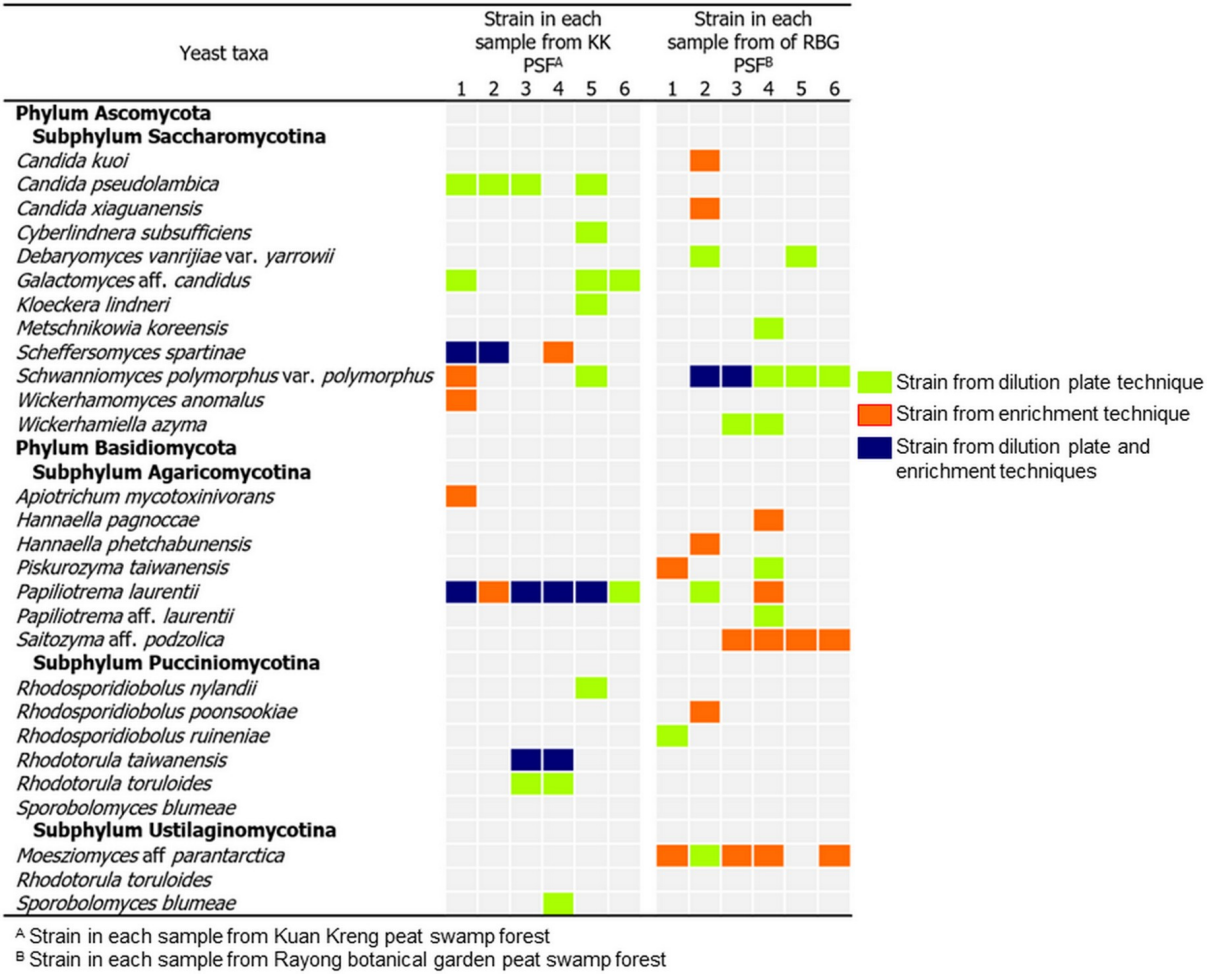
Yeast communities. Summary of the yeast communities of Kuan Kreng and Rayong Botanical Garden peat swamp forests isolated by the dilution plate and the enrichment techniques.

From the three samples of soil and the three samples of peat soil, 35 yeast strains were obtained using the two isolation techniques, the dilution plate (13 strains) and the enrichment (22 strains) techniques. On the basis of the D1/D2 region of the LSU rRNA gene sequence similarity analysis, the 32 yeast strains (91.4%) were identified to be 13 known yeast species in 12 genera (Table 3 and Figs 2–4). Among these, 13 strains were found to be six species in six genera of the phylum Ascomycota, namely *Candida pseudolambica* (*Pichia*/*Candida* clade), *Cyberlindnera subsufficiens*, *Hanseniaspora lindneri*, *Scheffersomyces spartinae*, *Schwanniomyces polymorphus* var. *polymorphus* and *Wickerhamomyces anomalus*. The other 19 strains were identified as seven species in six genera of the phylum Basidiomycota, namely, *Apiotrichum mycotoxinivorans*, *Papiliotrema laurentii*, *Saitozyma podzolica*, *Rhodosporidiobolus nylandii*, *Rhodotorula taiwanensis*, *Rhodotorula toruloides* and *Sporobolomyces blumeae*. In addition, there were three yeast strains that were closest to the type strain of *Galactomyces candidus* but differed by ten nucleotide substitutions out of 546 nucleotides in the D1/D2 region of the LSU rRNA gene. Consequently, they were considered as a potential new species closest to *Galactomyces* (S1 Table).

**Table 3:** Yeast communities.

Sample code	Isolated by dilution plate technique				Isolated by enrichment technique			
	No. of strain	Strain DMKU-	GenBank accession no.	Yeast species	No. of strain	Strain DMKU-	GenBank accession no.	Yeast species
**Kuan Kreng peat swamp forest**								
S01	4	SPS6-1	LC506234	*Wickerhamomyces anomalus*	4	ESS6-1	LC506246	*Scheffersomyces spartinae*
		SPS6-2	LC506235	*Scheffersomyces spartinae*		ESS6-3	LC506247	Potential new species closet to *Galactomyces candidus*
		SPS6-3	LC506236	*Papiliotrema laurentii*		ESS6-4	LC506248	*Papiliotrema laurentii*
		SPS6-4	LC506237	*Apiotrichum mycotoxinivorans*		ESS6-5	LC506249	*Candida pseudolambica*
S02	2	SPS7-1	LC506238	*Scheffersomyces spartinae*	2	ESS7-1	LC506251	*Scheffersomyces spartinae*
		SPS7-2	LC506239	*Papiliotrema laurentii*		ESS7-2	LC506252	*Candida pseudolambica*
S03	2	SPS8-1	LC506240	*Rhodotorula taiwanensis*	4	ESS8-1	LC506253	*Candida pseudolambica*
		SPS8-2	LC506241	*Papiliotrema laurentii*		ESS8-2	LC506254	*Papiliotrema laurentii*
						ESS8-3	LC506255	*Rhodotorula taiwanensis*
						ESS8-4	LC506256	*Rhodotorula toruloides*
S04	3	SPS9-1	LC506242	*Scheffersomyces spartinae*	4	ESS9-1	LC506257	*Papiliotrema laurentii*
		SPS9-2	LC506243	*Rhodotorula taiwanensis*		ESS9-3	LC506258	*Rhodotorula taiwanensis*
		SPS9-4	LC506244	*Saitozyma podzolica*		ESS9-4	LC506259	*Rhodotorula toruloides*
						ESS9-6	LC506260	*Sporobolomyces blumeae*
S05	1	SPS10-1	LC506245	*Papiliotrema laurentii*	8	ESS10-1	LC506261	Potential new species closet to *Galactomyces candidus*
						ESS10-3	LC506263	*Schwanniomyces polymorphus* var.
								*polymorphus*
						ESS10-4	LC506264	*Papiliotrema laurentii*
						ESS10-5	LC506265	*Rhodosporidiobolus nylandii*
						ESS10-7	LC506266	*Cyberlindnera subsufficiens*
						ESS10-8	LC506267	*Candida pseudolambica*
						ESS10-9	LC506268	*Hanseniaspora lindneri*
S06	0	-			3	ESS11-1	LC506269	*Papiliotrema laurentii*
						ESS11-3	LC506270	Potential new species closet to *Galactomyces candidus*
	**Total 12 strains**				**Total 26 strains**			
**Rayong Botanical Garden peat swamp forest**								
SR01	2	SPS12-1	LC420624	Potential new species closet to *Moesziomyces parantarctica*	1	ESS12-1	LC506405	*Rhodosporidiobolus ruineniae*
		SPS12-2	LC506392	*Piskurozyma taiwanensis*				
SR02	5	SPS13-1	LC506393	*Candida xiaguanensis*	s	ESS13-1	LC506406	*Schwanniomyces polymorphus* var. *polymorphus*
		SPS13-2	LC506394	*Hannaella phetchabunensis*				
		SPS13-3	LC506395	*Schwanniomyces polymorphus* var. *polymorphus*		ESS13-2	LC506407	*Schwanniomyces vanrijiae* var. *yarrowii*
						ESS13-3	LC506408	*Papiliotrema laurentii*
		SPS13-4	LC506396	*Rhodosporidiobolus poonsookiae*		ESS13-5	LC420628	Potential new species closet to *Moesziomyces parantarctica*
		SPS13-6	LC506397	*Starmerella kuoi*				
SR03	3	SPS14-1	LC506398	*Schwanniomyces polymorphus* var. *polymorphus*	2	ESS14-1	LC506409	*Schwanniomyces polymorphus* var. *polymorphus*
		SPS14-2	LC506399	*Saitozyma podzolica*		ESS14-2	LC506410	*Wickerhamiella azyma*
		SPS14-4	LC420625	Potential new species closet to *Moesziomyces parantarctica*				
SR04	4	SPS15-1	LC506400	*Papiliotrema laurentii*	5	ESS15-1	LC506411	*Metschnikowia koreensis*
		SPS15-2	LC420626	Potential new species closet to *Moesziomyces parantarctica*		ESS15-2	LC506412	*Schwanniomyces polymorphus* var. *polymorphus*
		SPS15-4	LC506401	*Saitozyma podzolica*		ESS15-3	LC506413	Potential new species closet to *Papiliotrema laurentii*
		SPS15-5	LC506402	*Hannaella pagnoccae*				
						ESS15-5	LC506414	*Piskurozyma taiwanensis*
						ESS15-6	LC506415	*Wickerhamiella azyma*
SR05	1	SPS16-4	LC506403	*Saitozyma podzolica*	2	ESS16-1	LC506416	*Schwanniomyces vanrijiae* var. *yarrowii*
						ESS16-2	LC506417	*Schwanniomyces polymorphus* var. *polymorphus*
SR06	2	SPS17-1	LC420627	Potential new species closet to *Moesziomyces parantarctica*	1	ESS17-1	s	*Schwanniomyces polymorphus* var. *polymorphus*
		SPS17-2	LC506404	*Saitozyma podzolica*				
	**Total 17 strains**				**Total 15 strains**			

Yeast communities from Kuan Kreng and Rayong Botanical garden secondary peat swamp forests.

The results showed that ascomycetous and basidiomycetous yeasts were found in equal proportions. In the present study, two isolation techniques were used. The results in Fig 3 showed that by using the dilution plate technique, seven yeast species comprising two species of Ascomycota and five species of Basidiomycota were obtained. Whereas ten species consisting of six species of Ascomycota and four species of Basidiomycota were obtained by the enrichment isolation technique. Among these, three species (*S*. *spartinae*, *P*. *laurentii* and *R*. *taiwanensis*) were found by both techniques.

### Yeasts from Rayong Botanical Garden peat swamp forest identified by molecular taxonomy and phylogenetic analyses

In total, 32 yeast strains were isolated from the six soil samples. These consisted of 17 strains obtained by the dilution plate technique and 15 strains by the enrichment technique. Among the 32 yeast strains, 26 strains (81.2%) were identified to be 13 known yeast species in 11 genera (Table 3 and Figs 2–4). Among the known species, 14 strains belonged to six species in five genera of the phylum Ascomycota, namely, *Starmerella kuoi*, *Candida xiaguanensis* (*Candida*/*Lodderomyces* clade), *Debaryomyces vanrijiae* var. *yarrowii*, *Metschnikowia koreensis*, *Sch*. *polymorphus* var. *polymorphus* and *Wickerhamiella azyma*, Twelve strains were found to belong to seven species in five genera of the phylum Basidiomycota; these were *Hannaella pagnoccae*, *Hannaella phetchabunensis*, *Piskurozyma taiwanensis*, *Papiliotrema laurentii*, *Sa*. *podzolica*, *Rhodosporidiobolus poonsookiae* and *Rhodosporidiobolus ruineniae*. In addition, six yeast strains (18.7%) showed sequences of the D1/D2 and ITS regions distinct from any known yeast species that was deposited in the GenBank database. These six strains were designated as two potential new species belonging to the genera *Moesziomyces* (five strains) and *Papiliotrema* (one strain) closest to *Moesziomyces parantarctica* and *P*. *laurentii*, respectively (Table 3). The number of basidiomycetous yeast strains (56.2%) was higher than that of ascomycetous yeasts (43.8%). By using the two isolation techniques, 10 species (three species of Ascomycota and seven species of Basidiomycota) were found by the dilution plate isolation technique (Table 3). While eight species (four species of Ascomycota and four species of Basidiomycota) were obtained by the enrichment technique. Among these species only four species, which were *Sch*. *Polymorphus* var. *polymorphus*, *P*. *taiwannensis*, *P*. *laurentii* and the potential new yeast species closest to *M*. *parantartica*, were found by both isolation techniques.

### Evaluation of antagonistic activity of yeasts against phytopathogenic fungi

Evaluation of the *in vitro* antagonistic activity of all 70 yeast strains isolated from the two PSFs against the seven fungal pathogens, comprising five species that cause rice diseases and three species that cause postharvest fruit diseases, revealed that only 13 yeast strains of seven species showed antagonistic activities. Eight yeast strain inhibited growth of at least fungal pathogen cause of rice diseases with high inhibition percentages (81.9–69.8%), whereas *H*. *lindneri* inhibited two rice pathogens by 84.4–72.8% (Table 4). Among these yeasts, *S*. *kuoi* DMKU-SPS13-6, *H*. *lindneri* DMKU ESS10-9 and *P*. *taiwanensis* DMKU-SPS12-2 showed high inhibition on *R*. *solani*. Three strains of *R*. *taiwanensis* (DMKU SPS8-1, DMKU ESS9-3 and DMKU SPS9-2) and one strain of *W*. *anomalus* (DMKU SPS6-1) were able to inhibit *C*. *lunata*. Only two strains, *H*. *lindneri* DMKU ESS10-9 and *S*. *spartinae* DMKU SPS9-3, inhibited *P*. *grisea*. Three strains (*S*. *kuoi* DMKU-SPS13-6, *H*. *lindneri* DMKU ESS10-9 and *P*. *taiwanensis* DMKU-SPS12-2) inhibited growth of *R*. *solani*. As for the fungal pathogens that cause postharvest fruit diseases, only *P*. *palmivora* was inhibited by four strains of *P*. *laurentii* (DMKU-SPS15-1, DMKU-ESS11-2, DMKU-ESS8-2, and DMKU-ESS6-4) (Table 4). No growth inhibition of *C*. *gloeosporioides*, *F*. *moniliforme*, *H*. *oryzae* or *L*. *theobromae* by any yeast strain was observed.

**Table 4:** Growth inhibition of fungal pathogens.

Yeast	Inhibition (%)			
	Rice pathogenic fungi			Postharvest pathogenic fungi
	*Rhizoctonia solani*	*Curvularia lunata*	*Pyricularia grisea*	*Phytophthora palmivora*
**Phylum Ascomycota**				
*Starmerella kuoi*				
DMKU-SPS13-6	70.1±0.43c	ND	ND	ND
*Hanseniaspora lindneri*				
DMKU ESS10-9	72.6±0.20b	ND	84.4±0.00a	ND
*Scheffersomyces spartinae*				
DMKU SPS9-3	ND	ND	81.9±1.10a	ND
*Wickerhamomyces anomalus*				
DMKU SPS6-1	ND	71.0±0.00b	ND	ND
**Phylum Basidiomycota**				
*Papiliotrema laurentii*				
DMKU-SPS15-1	ND	ND	ND	54.0±2.75b
DMKU-ESS11-2	ND	ND	ND	53.2±5.98b
DMKU-ESS8-2	ND	ND	ND	59.5±0.00ab
DMKU-ESS6-4	ND	ND	ND	59.5±4.12a
*Piskurozyma taiwanensis*				
DMKU-SPS12-2	76.2±0.20a	ND	ND	ND
*Rhodotorula taiwanensis*				ND
DMKU SPS8-1	ND	69.8±0.78b	ND	
DMKU ESS9-3	ND	71.9±0.08a	ND	ND
DMKU SPS9-2	ND	71.2±0.80ab	ND	ND

Growth inhibition of fungal pathogens cause of rice and postharvest durian fruit diseases by yeasts from Kuan Kreng and Rayong Botanical Garden peat swamp forests investigated by dual cultivation on PDA at 25°C for 3 days when tested with *Rhizoctonia solani* and 7 days when tested with *Curvularia lunata*, *Pyricularia grisea* and *Phytophthora palmivora*

Each sample was determined in triplicate.

ND: Not detected

a, b, and c indicated statistically significant (*P* < 0.05) intergroup difference

## Discussion

In this study, the yeast communities in soils and peat soils collected from two secondary PSFs were investigated. The dilution plate and the enrichment techniques were used for yeast isolation. The results demonstrated that all investigated samples contained yeasts. Yeasts in both phyla, Ascomycota and Basidiomycota, were found in the two PSFs. The numbers of strains in Ascomycota and Basidiomycota were equal in KK PSF, which agrees well with the result of the yeast community study in secondary PSF of Khanthuli PSF [38]. Whereas, the number of strains in Ascomycota was slightly lower than that in Basidiomycota in RBG PSF (Table 3). Among 26 species detected in the two secondary PSFs, only three species, namely *P*. *laurentii*, *S*. *podzolica* and *S*. *polymorphus* var. *polymorphus*, were found in both PSFs. This could be a consequence of differences in the physiochemical properties of the soils of the two forests. In addition, the plant communities of the two secondary PSFs were different, which could also affect the yeast communities. In the present study, two isolation techniques were performed. From KK PSF, the number of ascomycetous species obtained by the enrichment technique was higher (six species) than that by the dilution plate technique (two species). Whereas five and four basidiomycetous species were obtained by the dilution plate and the enrichment techniques, respectively. From RBG PSF, a higher total number of species was obtained by the dilution plate technique (ten species) than by the enrichment technique (eight species). The difference was the result of a difference in basidiomycetous species, the number of which found by the dilution plate technique was higher. By both isolation techniques, only five species (*Sch*. *polymorphus* var. *polymorphus*, *Pis*. *taiwanensis*, *P*. *laurentii*, *R*. *taiwanensis* and the potential new species closest to *Mo*. *parantarctica*) could be detected. Therefore, the result indicated that the isolation technique affected the assessment of yeast communities in the same way as was reported by Boonmak et al. [52]. However, it is not clear whether the enrichment isolation technique favors ascomycetous yeast isolation.

Not only from Thailand but also from tropical regions all over the world, there have been few reports on yeast communities of tropical PSFs. Jaiboon et al. [51] reported cultivable yeast species isolated by the enrichment technique from peat at different depths from To Daeng PSF. This forest, which is located in the southern Thailand, is the largest and most fertile primary PSF in the country. The vegetation in the To Daeng forest comprises a great diversity of plant species including trees, palm trees, climbing plants, shrubs, fern and mosses [51], and is very different from the plant communities in the KK and RBG secondary PSF. In To Daeng PSF, ten known yeast species, *C*. *subsufficiens*, *Debaryomyces fabryi*, *Meyerozyma guilliermondii*, *Saturnispora diversa*, *S*. *polymorphus* var. *africanus*, *Cryptococcus taiwanensis*, *Cutaneotrichosporon mucoides*, *P*. *flavescens*, *P*. *laurentii* and *R*. *mucilaginosa* and one potential new species in the genus *Papiliotrema* were found. Among the species detected in To Daeng PSF by Jaiboon et al. [51], only *P*. *laurentii* and *R*. *mucilaginosa* were also found in KK and RBG PSFs.

Recently, Boonmak et al. [38] studied yeast communities of both primary and secondary PSF of KK PSF. They reported that the plant communities in the primary area are more diverse than those in the secondary area. In the secondary area of Khanthuli PSF, they detected 11 known yeast species, namely, *Candida albicans*, *C*. *maltosa*, *C*. *tropicalis*, *C*. *pseudolambica*, *C*. *subsufficiens*, *Pichia kudriavzevii*, *G*. *candidus* and *G*. *geotrichum* in the phylum Ascomycota and *S*. *podzolica*, *P*. *laurentii* and *R*. *mucilaginosa* in the phylum Basidiomycota. In addition, two new species, *Cryptotrichosporon siamense* [53] and *Saturnispora kantuleensis* [54] were discovered. By comparison, in the present study, which was carried out in two secondary PSFs, only five species *viz*. *C*. *pseudolambica*, *C*. *subsufficiens*, *G*. *candidus*, *P*. *laurentii* and *S*. *podzolica* were found in KK and RBG PSF studies. Among these species, only two species (*P*. *laurentii* and *S*. *podzolica*) were species that were common to the three secondary PSFs. While three species, *C*. *pseudolambica*, *C*. *subsufficiens* and *G*. *candidus*, were found in Khanthuli and KK secondary PSFs, which are located in the southern Thailand. The result of the present study and the two previous studies [38, 55] demonstrated that only *P*. *laurentii* was found in all four PSFs. Therefore, this could be interpreted to mean that *P*. *laurentii* was the yeast species common to both primary and secondary tropical PSF areas. All of these results suggested that the differences in the yeast communities were influenced by the physicochemical characteristics of the soil or peat soil and the vegetation [38].

There are many reports of yeast communities in boreal PSFs, which are mainly formed from the dead *Sphagnum* mosses. For instance, Thormann et al. [19] studied yeast species isolated from peatlands in Saskatchewan, Canada and West Siberia, Russia. They consisted of seven ascomycetous yeast species (*Candida edax*, *Candida haemulonis*, *Candida zeylanoides*, *Diutina catenulata*, *Nadsonia starkeyi-henricii*, *Nakaseomyces delphensis* and *Yarrowia lipolytica*) and five basidiomycetous species (*Buckleyzyma aurantiaca*, *Farysia acheniorum*, *Naganishia albida*, *Solicoccozyma aeria* and *Trichosporon inkin*). Kachalkin and Yurkov [17] reported 15 yeast species isolated from *Sphagnum* mosses in the forest and the swamp biotopes in the Tver region, Russia. Of these, seven species belonged to the phylum Ascomycota, namely, *Aureobasidium pullulans*, *Dothidella* sp., *Candida friedrichii*, *Candida sphagnicola*, *C*. *zeylanoides*, *Candida* sp. and *Pichia membranifaciens*, and eight species in the phylum Basidiomycota, *viz*., *Cystofilobasidium capitatum*, *Filobasidium wieringae*, *Filobasidium magnum*, *Naganishia diffluens*, *Vishniacozyma victoriae*, *Cystobasidium lysinophilum*, *Sporobolomyces roseus* and *R*. *mucilaginosa*. In these two reports of yeasts from difference habitats in boreal PSFs situated in different locations, only *C*. *zeylanoides* was found in both. Comparing the yeast communities of PSF in Thailand, which is a tropical country, and results of the two studies in Canada and Russia, which are temperate countries, only three species (*P*. *laurentii* and *R*. *mucilaginosa* and *Sc*. *vanrijiae*) were found to be species common to all the places [17, 19, 38, 51]. Therefore, the difference of yeast communities could also be the consequence of their different locations, which resulted in different climates and different habitats for yeast.

Investigation of antagonistic activities of 70 yeast strains isolated from the two secondary PSFs was performed. We found eight strains of six species (*S*. *kuoi*, *H*. *lindneri*, *R*. *taiwanensis*, *S*. *spartinae*, *W*. *anomalus* and *Pis*. *taiwanensis*) showed strong inhibition of the growth of the three rice fungal pathogens (*C*. *lunata*, *P*. *grisea* and *R*. *solani*). In addition, four strains of *P*. *laurentii* exhibited inhibition on growth of *P*. *palmivora*, a fungal pathogen that causes rot disease in durian fruit (Table 4). Interestingly, species in both Ascomycota and Basidiomycota revealed antagonistic activities against fungal pathogens of plant and postharvest fruit. To our knowledge, the present report is the first report on the antagonistic activity of *S*. *kuoi*, *H*. *lindneri*, *R*. *taiwanensis* and *S*. *spartinae*. *W*. *anomalus* against *C*. *lunata*, which causes dirty panicle disease in rice, as well as of *P*. *laurentii* against *P*. *palmivora*, which causes fruit rot disease of durian. Therefore, these yeasts have potential to be biological control agents and should be subjects of further research and development.

Among yeast species reported to have antagonistic activities in the present study, *W*. *anomalus* (formerly *Pichia anomala*) has been reported to be an effective biocontrol agent against fungal pathogens causing postharvest diseases. For example, Aloui et al. [56] found that *W*. *anomalus* completely suppressed green mold disease of orange caused by *Penicillium digitatum*. Campos-Martínez et al. [57] demonstrated that *W*. *anomalus* was able to suppress the growth of *Colletotrichum acutatum*, a cause of anthracnose on avocado fruits. *W*. *anomalus* BS91 showed high biocontrol activity against *Monilinia fructigena* and *Monilinia fructicola*, which cause of brown rot on peach and plum fruit, respectively [58]. *W*. *anomalus* Disva 2 was effective in the reduction of brown rot caused by *Monilinia laxa* on sweet cherry [59]. In addition, *W*. *anomalus* has been reported to have potential for the control of gray mold decay caused by *B*. *cinerea* on strawberry and grape [40, 60]. Recently, Zhang et al. [61] reported the ability of *W*. *anomalus* to suppress growth of blue mold decay caused by *Penicillium expansum* on pears. In this study, *W*. *anomalus* DMKU SPS6-1 revealed strong inhibition of the growth of *C*. *lunata*, which causes dirty panicle disease of the rice plant. Several studies have demonstrated the efficacy of *P*. *laurentii* (formerly *Cryptococcus laurentii*) as a biological control agent active against a wide range of fungal pathogens cause of postharvest diseases in various fruits. For example, *B*. *cinerea*, *P*. *expansum* and *Rhizopus tolonifer* in peach fruits [62], *P*. *expansum* in jujube fruits [63], *B*. *cinerea* and *P*. *expansum* in pear fruits [64], *C*. *gloeosporioides* in mango fruits [65], *B*. *cinerea* in cherry tomato fruits [66] and *P*. *italicum* in grapefruit fruits [67]. Although *P*. *laurentii* has been reported to have antagonistic activities against several fungal pathogens, it has never been reported to have an antagonistic effect on *P*. *palmivora*, which causes fruit rot of durian. In the present study, four *P*. *laurentii* strains were capable of inhibiting the growth of *P*. *palmivora*.

## Conclusion

In the present study, we have described the yeast communities of the two secondary PSFs, KK and RBG PSFs, based on culture-dependent approaches. Yeasts belonging to the two phyla, Ascomycota and Basidiomycota, seemed to be distributed equally in KK PSF, while there were slightly fewer yeasts in Ascomycota in RBG PSF than in Basidiomycota. The differences in physicochemical characteristics of soils, isolation techniques, sampling location and plant communities likely affected the yeast communities. To investigate yeast diversity based on a culture dependent approach, we suggest that more than one technique be used for yeast isolation in order to receive better information on culturable yeast communities. In this study, we found yeast strains of seven species, each of which was able *in vitro* to inhibit the growth of at least one fungal pathogen causing a rice disease (*C*. *lunata*, *P*. *grisea* and *R*. *solani*) or one postharvest fruit disease (*P*. *palmivora*). Therefore, these yeasts are promising candidates for research and development of effective biocontrol measures for agricultural applications. Moreover, our findings suggest that the soils and peat soils in PSFs are sources from which antagonistic yeast can be obtained.

## Supporting information

S1 Table Potential new yeast species. Yeast strains of potential new yeast species isolated from Kuan Kreng and Rayong botanical garden peat swamp forests.
(PDF)
