# Supplementary material for: Yeast communities of secondary peat swamp forests in Thailand and their antagonistic activities against fungal pathogens cause of plant and postharvest fruit diseases
Source: PLoS One. 2020 Mar 16;15(3):e0230269. doi: 10.1371/journal.pone.0230269 (PMC7075701; doi:10.1371/journal.pone.0230269)
Supplement: S1 Table — Yeast strains of potential new yeast species isolated from Kuan Kreng and Rayong botanical garden peat swamp forests. (PDF) [file pone.0230269.s001.pdf]

S1 Table. Potential new yeast species.

Strain DMKU-	Closest species	Nucleotide substitution/ gap/total nt.		GenBank accession no. (D1D2/ITS)	Identification result
		D1/D2 region	ITS region		
Yeast of in Kuan Kreng peat swamp forest					
Phylum Ascomycota					
ESS 6-3	*Galactomyces candidus* CBS 772.71^T (U40118/ HE663404)	11-12/1/546	1-11/0-4/253	LC506247, LC506273	Potential new species closet to *Galactomyces candidus*
ESS10-1				LC506261, LC506276	
ESS11-3				LC506270, LC506278	
Yeast of Rayong botanical garden peat swamp forest					
Phylum Basidiomycota					
ESS15-3	*Papiliotrema* sp. DMKU-CE120 (LC178811)	1/0/568	4/3/478	LC506413, LC506419	Potential new species closet to *Papiliotrema laurentii*
	Cryptococcus sp. YS NB5 (AM397842)	1/0/555	-		
	Papiliotrema laurentii CBS 139^T (AF075469/LK023833)	2/0/568	6/2/425		
SPS12-1	*Moesziomyces parantarctica* CBS 10005^T (AB089357/AB089356)	2/0/560	35/29/649	LC420624, LC420629	Potential new species closet to *Moesziomyces parantarctica*
SPS14-4				LC420625, LC420630	
SPS15-2				LC420626, LC420631	
SPS17-1				LC420627, LC420632	
ESS13-5				LC420628, LC420633	

Yeast strains of potential new yeast species isolated from Kuan Kreng and Rayong botanical garden peat swamp forests
